# Patient and clinician beliefs about potential barriers to treatment of neuropathic pain for adolescents with sickle cell disease

**DOI:** 10.1002/jha2.829

**Published:** 2023-12-01

**Authors:** Matthew Rees, Holly Spraker‐Perlman, Raechyl Moore, Paul Lavoie, Linda Schiff, Jennifer M. Allen, Parul Rai, Doralina L. Anghelescu

**Affiliations:** ^1^ Department of Oncology St Jude Children's Research Hospital Memphis Tennessee USA; ^2^ Department of Hematology St. Jude Children's Research Hospital Memphis Tennessee USA; ^3^ Department of Pharmaceutical Sciences St. Jude Children's Research Hospital Memphis Tennessee USA; ^4^ Department of Psychology St. Louis Children's Hospital St Louis Missouri USA; ^5^ Department of Pediatric Medicine St. Jude Children's Research Hospital Memphis Tennessee USA

**Keywords:** barriers, beliefs, neuropathic pain, sickle cell disease, survey, treatment

## Abstract

Pain is the hallmark symptom causing morbidity for people with sickle cell disease (SCD) and may present as nociceptive, neuropathic, or mixed type pain. Neuropathic pain (NP) is underrecognized and undertreated in patients with SCD and is associated with decreased patient‐reported quality of life. Surveys were completed by clinicians caring for adolescents with SCD in the outpatient setting. SCD patients ages 1418 at increased risk of NP completed a patient‐facing survey at a scheduled clinic visit. Ninety‐four percent of responding clinicians agreed that NP significantly contributes to reported pain in SCD. Clinicians believed that NP medications are effective for reducing chronic pain (62%) and decreasing opioid utilization (44%). Clinician‐identified barriers to prescribing NP medications included concerns about medication adherence (82%), lack of pediatric guidelines for NP medications (70%), and perceived patient concern about side effects (65%). More than 1/3 (35%) of clinicians reported that they were not comfortable managing NP medications. Clinician‐identified barriers to referral to a pain management specialist included scheduling concerns (88%) and perceived patient/family lack of interest (77%). Most patients expressed willingness to take a medication for NP (78%), see a pain management specialist (84%), or learn more about nonpharmacologic interventions (72%), although most (51%) also reported some concerns about taking a medication for NP, citing insufficient knowledge (34%), and potential for side effects (32%). A minority of respondents (15%) worried about referral to a pain management specialist. Clinician and patient perspectives provide insights that may guide education efforts or other interventions to improve treatment of SCD‐related NP.

## Graphical abstract

Adolescents with SCD are amenable to interventions to manage neuropathic pain. Further education and research may help better understand and address potential barriers to management of neuropathic pain. Abbreviations: SCD, sickle cell disease.

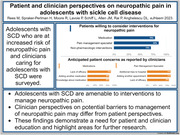

**Abbreviations**
MRI: magnetic resonance imaging
NP: neuropathic pain
SCD: sickle cell disease

## 1 INTRODUCTION

Pain is the hallmark symptom causing morbidity for people with sickle cell disease (SCD) and may present as nociceptive, neuropathic, or mixed type pain [1, 2]. Pain may also be either acute or chronic in nature, with chronic pain defined as pain that is present on most days over a period of at least 3 months [3]. Among many potential contributing factors, neuropathic pain (NP) is thought to play a pivotal role in the development of chronic pain through nervous system sensitization and dysregulation of neural pathway connectivity [4, 5, 6]. NP results from dysfunction of the central or peripheral somatosensory system, rather than tissue injury as in inflammatory or nociceptive pain [4, 5]. NP may manifest as hypersensitivity to thermal or tactile stimuli and be reported as a sensation of tingling, numbness, burning, or radiating pain. Prior studies have demonstrated hypersensitivity to thermal stimulation in patients with SCD, with a variety of mechanisms contributing to this hypersensitivity identified in murine models of SCD [7, 8, 9, 10]. One study also demonstrated differences in resting state neural connectivity by functional magnetic resonance imaging (MRI) in patients with SCD [6]. Together, these observations suggest a potential framework to understand a relationship between NP and the development of chronic pain in SCD and highlight the importance of better understanding the prevalence, manifestations, assessment, and treatment of NP in patients with SCD.

Risk factors for NP in adolescents and young adults with SCD include older age, female sex, hydroxyurea therapy, and increased frequency of acute care visits for pain [5, 11, 12]. While the association between hydroxyurea therapy and the presence of NP has been shown in multiple studies, this relationship has not been further elucidated. Some have proposed that hydroxyurea is merely a proxy for those with more severe disease manifestations [5, 12, 13]. Psychosocial factors such as psychiatric illness, lower socioeconomic status, lower educational attainment, and employment status have also been associated with increased prevalence of neuropathic pain in the general population or in other conditions. However, no such association has been evaluated to date in patients with SCD [14, 15, 16].

Despite the increasing understanding of the importance of NP in SCD, recent studies suggest that NP is still underrecognized and undertreated in patients with SCD and is associated with decreased patient‐reported quality of life [5, 12, 17, 18]. Studies have reported NP prevalence as high as 25%–40% in adolescent or young adult populations with SCD but a much lower percentage of patients received treatment directed at NP [5, 12, 17]. There are many factors implicated in the poor identification of NP in SCD. These include a lack of education among providers and a nascent understanding of the neurobiology of SCD‐related pain and of the relationships between acute, chronic, nociceptive, and neuropathic pain [1, 5]. Limitations in clinical assessment also play a key role, as there are no NP assessment tools validated specifically for the SCD population [19]. Quantitative sensory testing and functional MRI are primarily research tools that are not clinically available in most centers [1, 5]. However, several NP assessment tools have been validated in other populations and used in patients with SCD, including the painDETECT questionnaire, Leeds Assessment of Neuropathic Symptoms and Signs, and Douleur neuropathique en 4 questions (DN‐4) questionnaire [20, 21, 22, 23].

Furthermore, there is a paucity of data guiding management of NP in SCD, and pain medications commonly used for patients with SCD (acetaminophen, nonsteroidal anti‐inflammatory drugs, or various opioids) are often ineffective in treating NP. Therapies that are typically more effective in treating NP, such as gabapentinoids (gabapentin, pregabalin), methadone, or serotonin norepinephrine reuptake inhibitors, may not be frequently prescribed to treat SCD‐related pain and have not been well‐studied in children with SCD [1, 24]. Preclinical SCD murine data showed efficacy of gabapentin in reducing both acute and chronic pain [25], but confirmatory studies in humans are limited. One small randomized controlled trial in adults with SCD compared pregabalin to placebo for impact on self‐reported NP and quality of life, but limitations of small sample size, attrition, and short duration of the study limit meaningful conclusions [24, 26]. In children with SCD, one trial randomized patients presenting with acute vaso‐occlusive pain to gabapentin or placebo but did not include NP as a prerequisite for study inclusion or as a specific outcome measure [27]. However, case reports and anecdotal experience suggest that these interventions may alleviate some of the symptoms stemming from SCD‐related NP in children and adolescents [28, 29].

There are likely additional patient‐ and clinician‐related barriers to effective identification and management of SCD‐related NP, but these are poorly understood. We surveyed clinicians caring for patients with SCD and adolescent patients with SCD to better understand underlying attitudes and beliefs about NP and explore potential barriers to management of SCD‐related NP.

## 2 METHODS

Survey metrics were developed to separately assess clinician and patient attitudes and beliefs about SCD‐related NP and potential interventions to manage NP (Supplemental Appendix 1). Some survey questions were adapted from patient and clinician surveys developed by the Sickle Cell Disease Implementation Consortium [30]. Other questions were based on review of the literature and clinician experience. A multidisciplinary team consisting of physicians (in pediatric hematology, palliative care, and anesthesia/pain management), advanced practice providers, a nurse, a pharmacist, and a pediatric psychologist developed and reviewed survey items to determine appropriateness for use. Items were iteratively reviewed and edited for content and face validity. Survey instruments were reviewed and approved by the institutional review board for use as part of an ongoing quality improvement initiative to improve detection and treatment of NP in patients with SCD.

Clinicians surveyed included practicing Pediatric Hematologists, fellows in pediatric hematology/oncology, and advanced practice providers (nurse practitioners and physician assistants) in the hematology clinic at a tertiary care hospital specializing in pediatric hematology, oncology, and other serious illnesses affecting children. Surveys were sent electronically to clinicians prior to implementation of the quality improvement initiative to capture baseline data prior to any interventions.

Patients were considered for eligibility if they had a diagnosis of SCD (all genotypes), were ages 14–18 years, and had at least one previously identified risk factor for NP by chart review. These risk factors included female sex, current hydroxyurea therapy, or history of at least three acute care visits for pain in the preceding 12 months. Patients were excluded if they were younger than age 14, had no identified risk factors, were already receiving a medication for NP, or if they were receiving ongoing treatment by a pain management specialist. Children <14 years were excluded to mirror previous experience with the use of the painDETECT questionnaire in patients with SCD [5]. Eligible patients were asked to complete a brief patient‐facing survey along with a painDETECT questionnaire [21] and the Sickle Cell Pain Burden Inventory‐Youth (SCPBI‐Y) [31] at the time of a regularly scheduled clinic visit. Patient surveys were collected from April 2022 through February 2023 in the outpatient Hematology Clinic. Survey results were analyzed independent of results from painDETECT and SCPBI‐Y questionnaires. Only the survey results are presented in this study.

## 3 RESULTS

### 3.1 Clinician survey results

**FIGURE 1:**
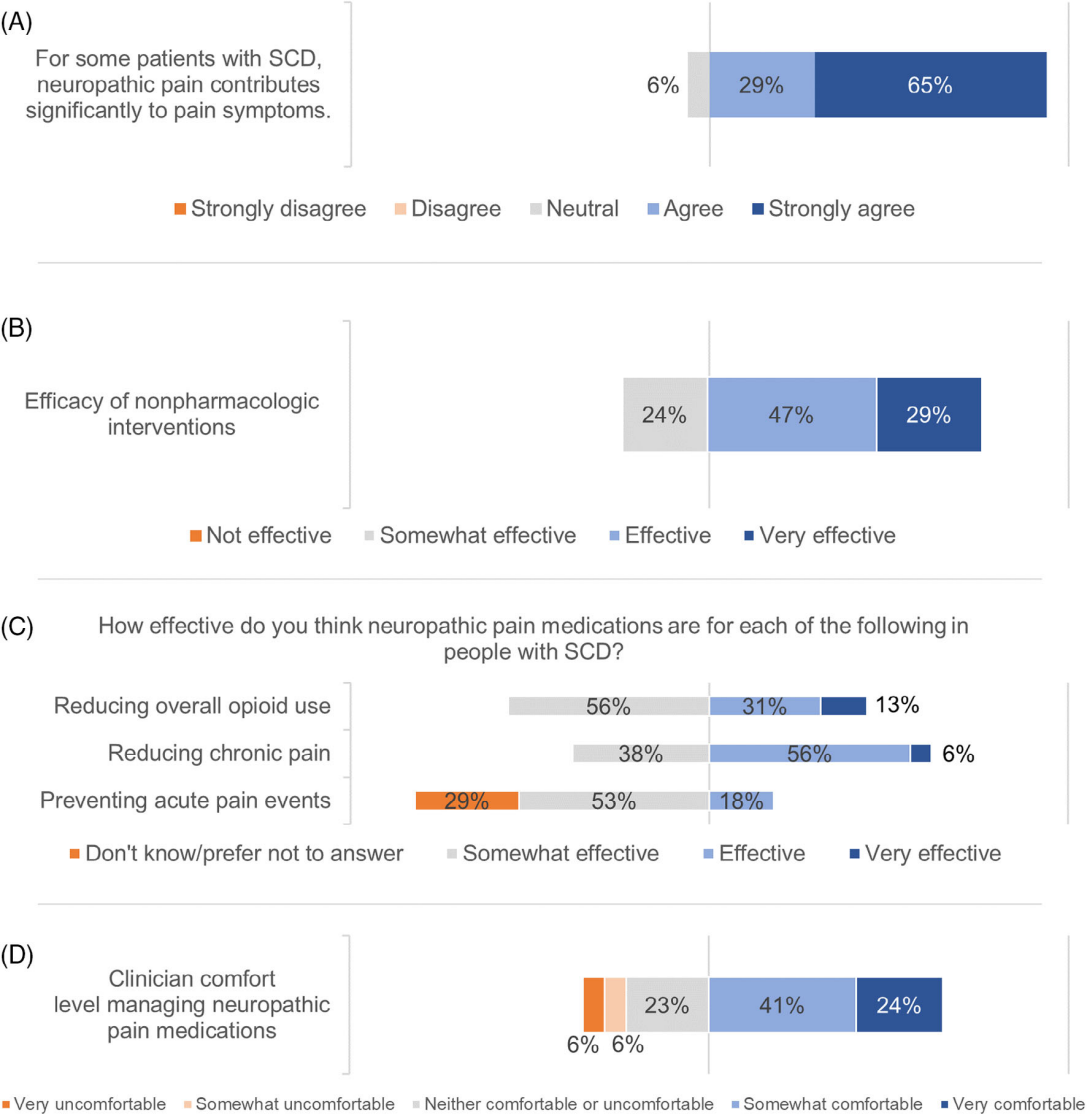
Clinician beliefs about neuropathic pain and potential interventions.

The clinician survey was sent electronically to 49 clinicians, and 17 clinicians completed the survey (response rate 35%). The respondents consisted of five advanced practice providers, five pediatric hematologists, and seven pediatric hematology/oncology fellows. Nearly all respondents (94%) agreed that NP contributes significantly to pain symptoms in some SCD patients (Figure 1A).

### 3.2 Clinician beliefs about nonpharmacologic and pharmacologic interventions for pain

Clinicians were asked about efficacy of nonpharmacologic interventions such as psychological modalities, physical therapy, and integrative medicine, and their efficacy in managing chronic pain for patients with SCD. A majority of the respondents believed that these non‐pharmacologic interventions were either very effective (29%) or effective (47%) for patients with SCD with chronic pain (Figure 1B).

More clinicians believed that NP medications are effective for reducing chronic pain (62%) and decreasing opioid use (44%), than those believing in their efficacy for preventing acute pain events (18%) (Figure 1C). While most clinicians responded favorably regarding their comfort managing NP medications, only one in four was very comfortable (24%) and more than one third (35%) of clinicians were not comfortable managing NP medications (Figure 1D).

### 3.3 Clinician‐identified barriers to interventions for NP

**FIGURE 2:**
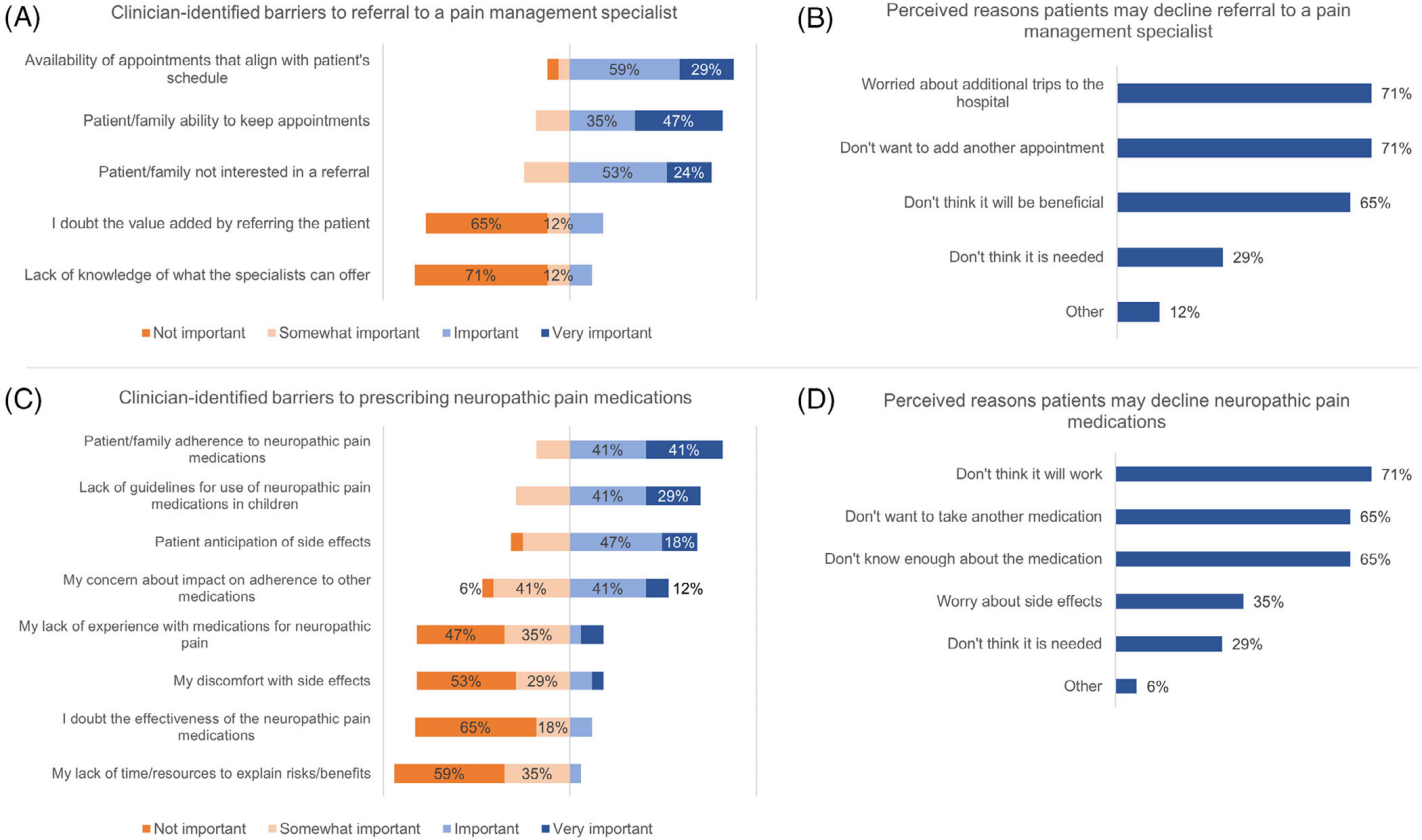
Clinician‐identified barriers and clinician perceptions of patient‐identified concerns regarding potential interventions.

When asked about referring a patient with possible NP to a pain management specialist for further evaluation and treatment, the most frequent clinician‐identified barriers to referral included scheduling concerns, related to both the availability of appointments at an appropriate time for the patient (88%), as well as the patient or family's ability to keep the appointment when scheduled (82%). Many also cited a perception that patients or families may not be interested in a referral to a pain management specialist (77%). However, most clinicians responded that their own perception of the potential value of a referral and their familiarity with what might be offered by a pain management specialist were not significant barriers to offering a referral when indicated (Figure 2A). Clinicians’ perceptions of potential patient concerns also highlighted scheduling and transportation issues. Clinicians were asked to consider reasons that a patient with evidence of NP might decline referral to a pain management specialist. Seventy‐one percent of clinicians cited patients’ concern about additional trips to the hospital or adding additional appointments to their schedule, and nearly two‐thirds (65%) worried that some patients may not think the referral would be beneficial (Figure 2B).

Clinician‐identified barriers to prescribing NP medications included concerns about patient adherence, lack of formal management guidelines, and potential side effects (Figure 2C). Eighty‐two percent of clinicians responded that concerns about patient or family adherence to NP medications would be a potential barrier to prescribing, while 53% reported a concern that prescribing a medication for NP may impact patient adherence with other prescribed medications. The lack of guidelines for use of NP medications in children was cited by 70%, and 65% reported that patient anticipation of side effects may be an additional deterrent.

Clinicians were asked to consider patients with evidence of NP and possible reasons patients may decline NP medications. The most cited belief surrounding patient refusal was that patients may not believe in the efficacy of NP medications for pain (71%) (Figure 2D). Nearly two‐thirds of clinicians felt that patients may not be interested in an additional medication (65%), and that patients’ lack of knowledge about the medications (65%) would be a barrier. Fewer clinicians reported beliefs that patients would decline a NP‐directed prescription based on concerns about side effects (35%) or due to the opinion that the medication was not necessary to adequately manage their pain (29%).

### 3.4 Patient survey results

A total of 104 patients completed all or part of the patient survey. Fifteen patients who did not meet eligibility criteria were erroneously given surveys at the time of the clinic visit and their responses were excluded from analysis due to one or more of the following: age <14 years (seven patients), lack of identified risk factors for NP (six patients), current prescription of NP medications (three patients), or ongoing management by a pain specialist (two patients). Seven eligible patients who completed the painDETECT questionnaire only (and not additional survey items) were also excluded. Eighty‐two patients who completed all or part of the survey and met the inclusion criteria were included in this analysis (Table 1). When an eligible patient completed the survey at more than one visit, the initial survey was used, and other subsequent responses were excluded.

**TABLE 1:** Demographics of included participants.

Sex (%)	Male 30 (37%)	Female 52 (63%)	All Participants (*n* = 82)
**Age** (median, range)	15.8 (14.0–18.4)	16.0 (14.0–18.6)	16.0 (14.0–18.6)
**Sickle cell genotype**	***N* (%)**	***N* (%)**	***N* (%)**
*HbSS*	25 (83%)	30 (56%)	55 (67%)
*HbSC*	2 (7%)	17 (33%)	19 (23%)
*HbSβ^0^‐thalassemia*	3 (10%)	0	3 (4%)
*HbSβ^+^‐thalassemia*	0	5 (9%)	5 (6%)
**Additional risk factors**			
*Hydroxyurea currently prescribed*	30 (100%)	34 (65%)	64 (78%)
*≥3 acute care visits for pain in preceding 12 months*	6 (20%)	8 (15%)	14 (17%)

Abbreviations: HbSC, hemoglobin SC disease; HbSS, hemoglobin SS disease; HbS*β^+^*‐thalassemia, hemoglobin S with *β^+^* thalassemia; HbSβ^0^‐thalassemia, hemoglobin S with β^0^ thalassemia.

The median age for the cohort was 16.0 years (range 14.0–18.6), and 63% of respondents were female (Table 1). Fifty percent of patients had one identified risk factor for NP, and 41% and 9% had two and three risk factors, respectively. Current hydroxyurea prescription was the most common risk factor (78% of patients), followed by female sex (63%) and presence of ≥3 acute care visits for pain in the preceding 12 months (17%).

**FIGURE 3:**
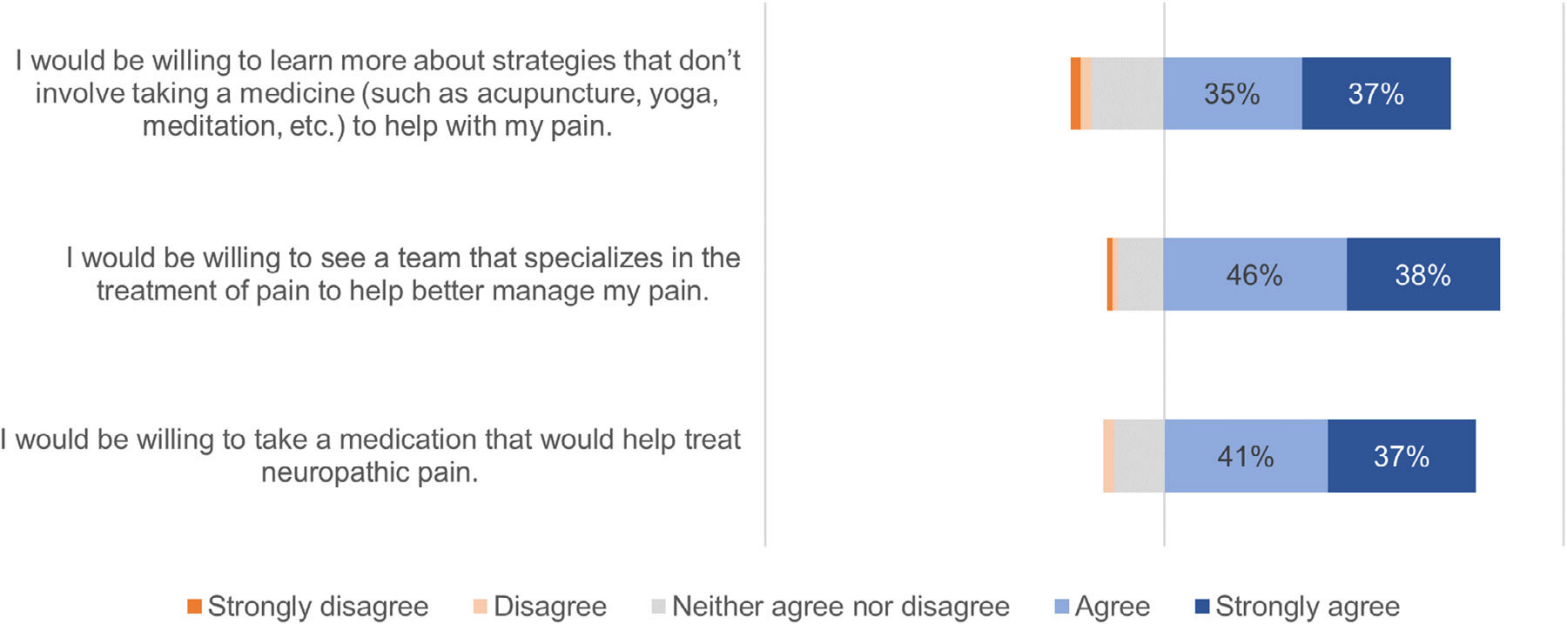
Patient attitudes regarding potential interventions for neuropathic pain.

Most patients expressed willingness to take a medication for NP (78%), see a pain management specialist (84%), and learn more about nonpharmacologic interventions, such as acupuncture, yoga, and meditation (72%; Figure 3) if recommended by their care team. Most patients who did not respond affirmatively were neutral, and only a very small percentage of patients expressed unwillingness to consider these options (3%, 2%, and 6%, respectively).

### 3.5 Patient‐identified concerns about NP treatment

**FIGURE 4:**
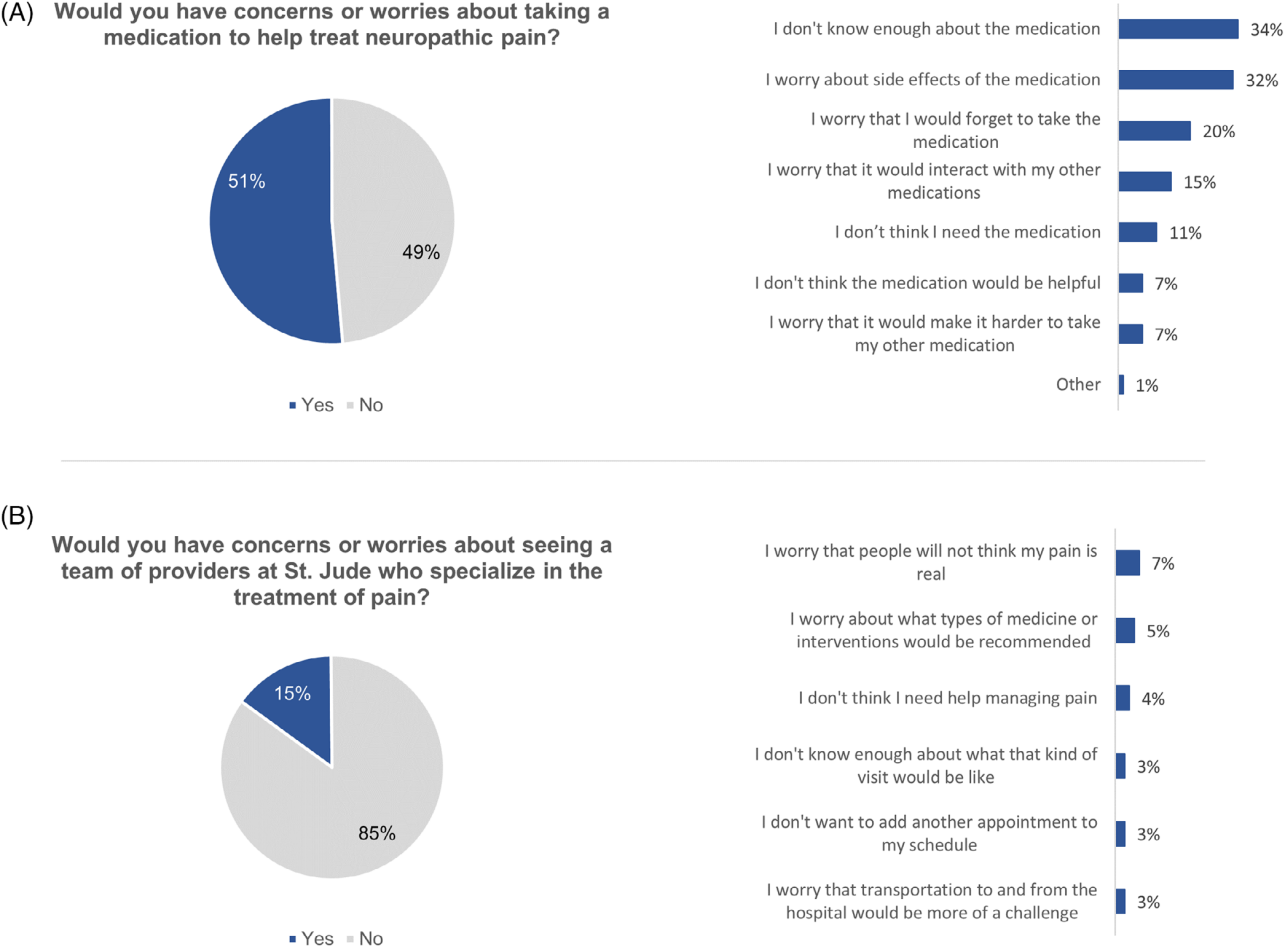
Patient concerns regarding use of medications or referral to pain management specialist for neuropathic pain.

Concerns about taking a medication for NP were much more common than fears about seeing a pain management specialist. Approximately half of the patients responding (51%) indicated some concerns about taking a medication for NP, compared to fewer than 1 in 6 patients (15%) reporting concerns about seeing a team specializing in pain management (Figure 4). The most common concerns about medications were insufficient knowledge about the medication (34% of all patients) and concern about potential side effects (32%; Figure 4A). Some participants also cited concern about forgetting to take the medication (20%) or potential interactions with other medications (15%). Notably, concerns about efficacy (7%) and potential impact on adherence to current medications (7%) were not commonly cited by patients. One patient commented that they would worry about needing additional needle pokes for monitoring laboratory draws.

Among the smaller subset of patients reporting concerns about seeing a pain management specialist, the most common concern was needing to legitimize their pain to another clinical team (Figure 4B). This concern was cited by five patients (7% of all respondents). Worry about what types of medications or treatments would be recommended (5%) was the next most frequently cited concern (Figure 4B). Concerns about scheduling additional appointments (3%) or arranging transportation (3%) were not prevalent.

## 4 DISCUSSION

This report provides insights into patient and clinician perspectives about NP in patients with SCD at a large, pediatric hematology/oncology‐specific center. An understanding of these attitudes and beliefs may help guide efforts to improve detection and treatment of NP in this population by identifying and addressing potential barriers. While high‐quality evidence establishing the true prevalence of NP in patients with SCD, its optimal treatment, and potential treatment outcomes is lacking, current evidence shows that NP in patients with SCD is underrecognized, undertreated, and correlates with lower ratings of patient‐reported quality of life [5, 12, 17, 18]. Improved diagnosis and management of neuropathy may decrease the overall symptom burden for these patients and lead to improved quality of life.

We did not identify prior reports of patient or clinician perspectives specific to management of NP in SCD. Several studies have collected perspectives on general pain management or management of acute pain in SCD, highlighting common themes such as the importance of trust in the clinician‐patient relationship, frequent underestimation of pain, clinician concerns about adherence and drug‐seeking behaviors, stigma associated with certain medications, and concerns about side effects and risk of addiction if patients are prescribed opioids [32, 33, 34, 35]. One study of perspectives on chronic pain in SCD noted similar concerns regarding the risk of opioid addiction and withdrawal, suggesting that some clinicians may be more hesitant to prescribe opioids to patients with SCD than other patients with chronic pain [36]. Studies in non‐SCD patient populations with NP have demonstrated that patients are frequently dissatisfied with their ability to adequately manage their NP [37, 38]. Identified concerns or barriers related to management of NP in non‐SCD patient populations include inadequate access to care or pain management expertise, fear of addiction, and potential adverse effects of medications [39, 40]. While none of these studies specifically addressed SCD‐related NP, NP is typically experienced as part of the overall pain experience for patients with SCD, and attitudes and perceptions about other aspects of SCD‐related pain likely influence and provide important context for understanding beliefs about NP, including those reported here. Our study does not specifically assess concerns about addiction, but there remains considerable overlap between the perspectives prevalent in general pain management in SCD and the beliefs expressed by patients and clinicians in our cohort.

In general, clinicians viewed NP as an important symptom for at least some patients with SCD and felt that interventions to manage NP, including NP medications, referral to a pain management specialist, and nonpharmacologic interventions, would be effective. Importantly, clinician concerns about efficacy or value of NP medications or referral to a pain management specialist were not prevalent, but clinicians were concerned that patients’ perceptions of efficacy or value may lead to refusal of these recommended interventions. These concerns did not align with patient responses, as most patients were willing to consider interventions offered by the care team, including medications, referral to a specialist, and nonpharmacologic interventions. Only a small number of patients reported concern that a medication might not be helpful in managing their pain, indicating that clinicians may be overestimating patients’ concerns in this area.

The reasons for these discrepant views are not explored further in this study and merit close attention in subsequent studies. Given the tendency of clinicians to underestimate the overall pain experience of patients, part of the discrepancy may be related to an underappreciation of patients’ willingness to seek relief. Further, NP in SCD is not commonly an isolated symptom, but rather often occurs in the context of chronic pain and other symptoms that may be difficult to ameliorate and for which opioids play a prominent role in management. In this context, concerns relating to drug‐seeking behaviors or opioid addiction may unintentionally cloud a clinician's perceptions of patients’ willingness to consider alternatives to opioids. It is also possible that the survey overstates patient willingness to consider a new medication or referral in a theoretical scenario with few details, where patient responses may be influenced by an overestimation of the potential efficacy of various interventions. At least, the discrepancy highlighted by these results provides opportunities to educate providers about patient willingness to consider these interventions, and to explore opportunities for improved dialogue between clinicians and patients to better understand and amplify patient perspectives.

There was more agreement between clinician perceptions and patient responses regarding baseline knowledge and potential side effects of the medications. Nearly two‐thirds of clinicians cited patient concern about side effects and lack of knowledge about a medication as potential barriers, and these two concerns were the most frequently cited by patients. This finding aligns with concerns identified in previous studies regarding pain management in patients with SCD [32, 33, 34, 35, 36]. Clinicians should be aware that lack of education regarding medication mechanism, utility, and potential side effects are potential barriers to patient use, and plan for thorough, patient‐facing conversations to assuage these fears. In addition, patient‐accessible education materials are needed to readily explain the concepts of NP and potential risks and benefits of treatment. The high percentage of patients reporting willingness to take a medication for NP combined with the high percentage of patients also expressing some concerns or worries about taking the medication may indicate that patients have appropriate reservations or questions, but that these reservations may be resolved if adequately addressed by the clinician.

Several concerns cited by clinicians were secondary and related to implementation or logistics of the intervention, such as concerns about transportation or availability of appointments in the case of referral to a specialist, as well as concerns about patient adherence (either with the NP medication or a secondary effect on adherence with other prescribed medications). These concerns were generally not as prevalent in patient responses, and some of them may be amenable to focused efforts to more fully align clinical operations and resources with patient needs. Future efforts could quantitatively track adherence to medications (in terms of pharmacy dispensing and/or self‐reported use) as well as patient attendance at pain management, integrative medicine, and follow up appointments.

An important observation in these survey results was the relatively low level of comfort among clinicians in managing NP medications. This correlates with a frequently cited concern identified by clinicians of a lack of published guidelines for the use of NP medications in children. Further study is needed to determine optimal methods of assessment, diagnosis, and management of NP in younger children and in patients with SCD, including randomized controlled trials of NP medications in children and adolescents with SCD. Such research may lead to development of pediatric‐ and SCD‐specific guidelines which, along with targeted education of clinicians, may help improve clinician comfort in this area.

Patient willingness to consider nonpharmacologic interventions such as yoga, acupuncture, and meditation highlights an important area for further research and opportunities to enhance care. Two recent systematic reviews suggested that various non‐pharmacologic complementary or alternative approaches may be effective in reducing intensity and frequency of pain in children with SCD [41, 42]. Other studies have described successful implementation of integrative medicine clinics for patients with SCD, highlighting high rates of patient satisfaction, acceptance of integrative therapies, and improved follow up following implementation of these clinics [43, 44]. Future research can help further define effectiveness of these interventions for different types of pain and determine optimal approaches for combining both pharmacologic and nonpharmacologic treatment modalities in SCD.

There are several factors that limit generalizability of our results to the SCD population at large. Participating clinicians shared a multidisciplinary perspective based on their experience caring for patients with SCD in the outpatient setting; nevertheless, the sample was relatively small. The survey was designed to help guide education and improve outpatient SCD pain management efforts at our institution, rather than to produce generalizable results across a larger population. The patient and clinician participants represent a specialized pediatric hematology/oncology children's hospital with a large population of sickle cell patients (ages 0–18) and a pediatric hematology‐specific team of clinicians. Here, a multidisciplinary pain management team routinely works closely with the Hematology team in providing care to mutual patients. Many centers may not have a robust team of physicians, nurses, physical therapists, psychologists, and integrative medicine specialists to provide services as queried in this study. In our setting, concerns about patients’ access to subspecialty care, financial burdens of care, and transportation may not be as pronounced due to resources available at our hospital. Of note, there is no onsite emergency department at our institution and patients with acute pain episodes are commonly managed in an acute care clinic. Because of this workflow, emergency medicine clinicians were not included in the survey population, representing an important perspective in the management of SCD‐related pain that was not captured in these responses.

Further, as anticipated, given inclusion criteria, the cohort surveyed is predominantly female. Inclusion of female sex as a risk factor for NP meant that all female patients with SCD age 14 years or older were eligible to participate even in the absence of other risk factors. On the other hand, male participants (≥14 years old) were likely skewed towards a more severe disease phenotype as they required at least one risk factor for NP to be eligible to participate. This might explain the higher percentages of male participants with HbSS genotype, on hydroxyurea treatment, and with an increased number of acute care visits for pain. Previous reports have also identified older age as an important risk factor in NP, and our study population is younger compared to other studies assessing risk of NP where the older age may confer a higher risk of NP [4, 6]. Patients who had already been identified as having NP or following with the team of pain specialists were excluded.

This study highlights several areas for potential improvement in the management of SCD‐related NP. Patient education is a routine component of SCD‐related care in the pediatric population. Expanding educational materials and interventions to address NP‐related concepts, such as potential risks and benefits of NP medications, availability of nonpharmacologic interventions, and the role of a multidisciplinary approach to pain management, can empower patients to engage in informed shared decision‐making with clinicians. Clinician education can ensure familiarity and comfort with various treatment options for SCD‐related NP and help improve understanding of patient perspectives related to NP and pain management in general. The identified gaps in clinician and patient perspectives highlight the need to facilitate conversations between clinicians and patients, both on an individual level and with larger communities, to dispel misconceptions and ensure a unified approach in assessing and managing symptoms. The gaps also suggest that clinician education should include discussion of potential biases that may influence decision‐making around offering certain interventions. Further research is needed to better understand the reasons for this gap in perspectives, as well as the optimal methods of detecting and treating SCD‐related NP.

Overall, patients at our institution are generally willing to consider interventions to manage NP, and clinicians recognize the importance of NP and believe interventions can be effective for these patients. Effective identification and management of NP symptoms in patients with SCD will depend on understanding patient‐ and clinician‐identified barriers and implementing efforts to appropriately address these barriers.

## AUTHOR CONTRIBUTIONS

All authors contributed to the study design, data collection, analysis, and/or manuscript preparation in a significant way. All authors reviewed and agreed upon the content of the manuscript.

## CONFLICT OF INTEREST STATEMENT

Matthew Rees received support for travel and lodging expenses to attend an educational conference by Creative Educational Concepts. The conference was supported by a grant from Jazz Pharmaceuticals. No other authors declared any conflict of interest.

## FUNDING INFORMATION

The authors received no specific funding for this work.

## ETHICS STATEMENT

The study was determined to meet criteria for nonhuman subjects research by the St. Jude Children's Research Hospital Institutional Review Board (FWA00004775).

## PATIENT CONSENT STATEMENT

Participation in the survey was optional and was determined by the St. Jude Children's Research Hospital to meet criteria for nonhuman subjects’ research. Informed consent was not required for quality improvement activities.

## CLINICAL TRIAL REGISTRATION

The study was determined to meet criteria for nonhuman subjects research by the St. Jude Children's Research Hospital Institutional Review Board and was not registered as a clinical trial.

## Supporting information

Supporting Information

## Data Availability

The data that support the findings of this study are available from the corresponding author upon reasonable request.
