# Supplementary material for: Patient and clinician beliefs about potential barriers to treatment of neuropathic pain for adolescents with sickle cell disease
Source: EJHaem. 2023 Dec 1;5(1):11–20. doi: 10.1002/jha2.829 (PMC10887355; doi:10.1002/jha2.829)
Supplement: Supplementary file 1 — Supporting Information [file JHA2-5-11-s001.docx]

# Supplemental Appendix 1

# Provider survey:

Pain is a common complication for patients with sickle cell disease (SCD). This can include acute and chronic pain and may include elements of both nociceptive and neuropathic pain. Nociceptive pain results from tissue injury and stimulation of pain receptors. Neuropathic pain results from injury or dysfunction of the somatosensory nervous system, including both the central and peripheral nervous system.

Several screening tools have been developed to identify patients with neuropathic pain. The painDETECT questionnaire is a brief, validated tool with reported sensitivity of 85% and specificity of 80% in detecting neuropathic pain1. It has previously been used in patients with SCD as young as 14 years old.

Pain medications that are commonly used for patients with SCD (acetaminophen, NSAIDs, opioids) are often ineffective in treating neuropathic pain, when present. Other medications or therapies should be considered for patients with neuropathic pain.

In answering the following questions, please consider potential interventions for patients who have a painDETECT score indicative of neuropathic pain:

- Treatment with neuropathic pain medications, such as gabapentin or pregabalin, typically taken by mouth 2-3 times per day
- Referral to a pain specialist to further assess pain symptoms and prescribe appropriate treatment
- Additional nonpharmacologic interventions such as psychology, physical therapy, and integrative medicine

1. For some patients with SCD, neuropathic pain contributes significantly to pain symptoms. *(Answer choices: Strongly disagree, Disagree, Neutral, Agree, Strongly agree, Don’t know/Prefer not to respond)*
2. Considering patients who have a painDETECT score indicative of neuropathic pain, what do you think would be the most common reasons patients/families might refuse neuropathic pain medications? (*Select up to 3)*
   1. Don’t know enough about the medication
   2. Don’t think it is needed
   3. Don’t think it will work
   4. Don’t want to take another medicine
   5. Worry about side effects
   6. Other, please specify:
   7. Don’t know/Prefer not to respond
3. Sometimes providers do not initiate a therapy even though its use might be indicated. Considering patients who have a painDETECT score indicative of neuropathic pain, how important would each of the following reasons be in your decision to prescribe medications for neuropathic pain? *(Answer options: Not important, Somewhat important, Important, Very important, Don’t know/Prefer not to respond)*
   1. Patient/family adherence to neuropathic pain medications
   2. My concern that adding a neuropathic pain medication would impact adherence to medications already prescribed
   3. Patient anticipation of side effects
   4. My discomfort with side effects
   5. My lack of experience with medications for neuropathic pain
   6. Doubt the effectiveness of the neuropathic pain medications
   7. My lack of time/resources to adequately explain risks/benefits
   8. There is a lack of formal guidelines for use of neuropathic pain medications in children
4. What resources or opportunities might help facilitate patient/family willingness to take neuropathic pain medications? *(Free text)*
5. What is your comfort level in managing neuropathic pain medications? *(Answer options: Very uncomfortable, Somewhat uncomfortable, Neither comfortable or uncomfortable, Somewhat comfortable, Very comfortable, Don’t know/Prefer not to respond)*
6. How effective do you think neuropathic pain medications are for preventing acute pain events in people with sickle cell disease? *(Answer options: Very effective, Somewhat effective, Effective, Not effective, Don’t know/Prefer not to respond)*
7. How effective do you think neuropathic pain medications are for reducing chronic pain in people with sickle cell disease? *(Answer options: Very effective, Somewhat effective, Effective, Not*

*effective, Don’t know/Prefer not to respond)*

1. How effective do you think neuropathic pain medications are for reducing overall opioid use in people with sickle cell disease? *(Answer options: Very effective, Somewhat effective, Effective, Not effective, Don’t know/Prefer not to respond)*
2. Considering patients who have a painDETECT score indicative of neuropathic pain, what do you think would be the most common reasons patients/families might refuse a referral to a team specializing in pain management? (*Select up to 3)*
   1. Don’t think it is needed
   2. Don’t think it will be beneficial
   3. Don’t want to add another appointment to their schedule
   4. Worried they might have to make additional trips to the hospital for appointments
   5. Other, please specify:
   6. Don’t know/prefer not to respond
3. Sometimes providers do not initiate a therapy even though its use might be indicated. Considering patients who have a painDETECT score indicative of neuropathic pain, how important would each of the following reasons be in your decision to refer the patient to a team specializing in pain management? *(Answer options: Not important, Somewhat important, Important, Very important, Don’t know/Prefer not to respond)*
   1. Patient/family not interested in a referral
   2. My concern about availability of appointments that align with the patient’s other scheduled appointments
   3. Patient/family ability to keep appointments
   4. Doubt the value added by referring the patient
   5. My lack of experience/knowledge of what the team of specialists can offer
4. What resources or opportunities might help facilitate patient/family acceptance of a referral to a team specializing in pain management? *(Free text)*
5. Some patients with chronic pain benefit from additional nonpharmacologic interventions such as psychology, physical therapy, and integrative medicine. How effective do you think these interventions are for people with sickle cell disease? *(Answer options: Very effective, Somewhat effective, Effective, Not effective, Don’t know/Prefer not to respond)*

References:

1. Freynhagen R, Baron R, Gockel U, Tölle TR. painDETECT: a new screening questionnaire to identify neuropathic components in patients with back pain. *Current Medical Research and Opinion*. 2006. 22:10, 1911-1920.

# Patient survey:

Pain is a common problem for people who have sickle cell disease. There are multiple types of pain that may contribute to the overall pain that a person experiences. One type of pain can come from damage or irritation of your nerves. This is called neuropathic pain. People may use words like burning, shooting, or “pins and needles” to describe neuropathic pain.

Research has shown that neuropathic pain may contribute to the overall pain experienced by some people with sickle cell disease. In some cases, medications or other treatments may help treat this kind of pain.

This survey asks some questions about neuropathic pain and possible treatments for neuropathic pain. It is completely voluntary. You can choose whether you want to complete this survey or not. If you choose not to complete the survey, you will still receive the same care that you would receive if you completed the survey. Your personal information will not be associated with any of your answers on this survey.

1. If my doctors thought that it would help me:
   1. I would be willing to take a medication that would help treat neuropathic pain. *(Answer choices: Strongly disagree, Disagree, Neutral, Agree, Strongly agree, Don’t know/Prefer not to respond)*
   2. I would be willing to see a team that specializes in the treatment of pain to help better manage my pain. *(Answer choices: Strongly disagree, Disagree, Neutral, Agree, Strongly agree, Don’t know/Prefer not to respond)*
   3. I would be willing to learn more about strategies that don’t involve taking a medicine (such as acupuncture, yoga, meditation, etc.) to help with my pain. *(Answer choices: Strongly disagree, Disagree, Neutral, Agree, Strongly agree, Don’t know/Prefer not to respond)*
2. Would you have concerns or worries about taking a medication to help treat neuropathic pain?

*(Answer choices: Yes, No)*

- 1. If yes, what concerns or worries do you have?
     1. I don’t know enough about the medication
     2. I don’t think I need the medication
     3. I don’t think the medication would be helpful
     4. I worry about side effects of the medication
     5. I worry that I would forget to take the medication
     6. I worry that it would make it harder to take my other medications
     7. I worry that it would interact with my other medications
     8. Other, please specify:

1. Would you have concerns or worries about seeing a team of providers at St. Jude who specialize in the treatment of pain? *(Answer choices: Yes, No)*
   1. If yes, what concerns or worries do you have?
      1. I don’t know enough about what that kind of visit would be like
      2. I don’t think I need more help managing pain
      3. I don’t want to add another appointment to my schedule
      4. I worry that transportation to and from the hospital would be more of a challenge than it is now
      5. I worry about what types of medicine or interventions would be recommended
      6. I worry that people will not think my pain is real
      7. Other, please specify:
